# Role of sex in lung cancer risk prediction based on single low-dose chest computed tomography

**DOI:** 10.1038/s41598-023-45671-6

**Published:** 2023-10-30

**Authors:** Judit Simon, Peter Mikhael, Ismail Tahir, Alexander Graur, Stefan Ringer, Amanda Fata, Yang Chi-Fu Jeffrey, Jo-Anne Shepard, Francine Jacobson, Regina Barzilay, Lecia V. Sequist, Lydia E. Pace, Florian J. Fintelmann

**Affiliations:** 1 Division of Thoracic Imaging and Intervention, Department of Radiology, Massachusetts General Hospital, 55 Fruit Street, Boston, MA 02114 USA; 2 Harvard Medical School, Boston, MA USA; 3 Department of Electrical Engineering and Computer Science, Massachusetts Institute of Technology, Cambridge, MA USA; 4 Jameel Clinic, Massachusetts Institute of Technology, Cambridge, MA USA; 5 Department of Medicine, Brigham and Women’s Hospital, Boston, MA USA; 6 Department of Surgery, Massachusetts General Hospital, Boston, MA USA; 7 Division of Thoracic Imaging, Department of Radiology, Brigham and Women’s Hospital, Boston, MA USA; 8 Department of Medicine, Massachusetts General Hospital, Boston, MA USA

**Keywords:** Cancer screening, Lung cancer

## Abstract

A validated open-source deep-learning algorithm called Sybil can accurately predict long-term lung cancer risk from a single low-dose chest computed tomography (LDCT). However, Sybil was trained on a majority-male cohort. Use of artificial intelligence algorithms trained on imbalanced cohorts may lead to inequitable outcomes in real-world settings. We aimed to study whether Sybil predicts lung cancer risk equally regardless of sex. We analyzed 10,573 LDCTs from 6127 consecutive lung cancer screening participants across a health system between 2015 and 2021. Sybil achieved AUCs of 0.89 (95% CI: 0.85–0.93) for females and 0.89 (95% CI: 0.85–0.94) for males at 1 year, p = 0.92. At 6 years, the AUC was 0.87 (95% CI: 0.83–0.93) for females and 0.79 (95% CI: 0.72–0.86) for males, p = 0.01. In conclusion, Sybil can accurately predict future lung cancer risk in females and males in a real-world setting and performs better in females than in males for predicting 6-year lung cancer risk.

## Introduction

Lung cancer is the leading cause of cancer mortality globally and is projected to remain a major cause of death for several decades^1,2^. Stage at the time of lung cancer diagnosis is the most important predictor of prognosis and early detection remains essential. In the National Lung Screening Trial (NSLT) and the NELSON study, early detection of small, asymptomatic lung tumors by low-dose chest computed tomography (LDCT) screening led to a significant reduction in lung cancer-specific mortality^3,4^. Since then, many countries have implemented large-scale LDCT screening, using eligibility criteria based largely on age and cigarette smoking history. The United States Preventive Services Task Force (USPSTF) recommends annual LDCT screening for individuals aged 50 to 80 years, who have a 20-pack-year smoking history and currently smoke or have quit smoking within the past 15 years^5^.

Sex is an important variable to consider in lung cancer screening programs since females have different risk profiles and prognoses compared to males^6–12^. There are also concerns that current screening criteria may be less applicable to females, since females smoke less when compared with males^13,14^. Over the last decades, the incidence of lung cancer has decreased far more slowly in females compared to males^15,16^. However, females remain underrepresented in lung cancer research^11,17–20^.

Researchers at Massachusetts General Hospital (MGH) and Massachusetts Institute of Technology (MIT) have developed and validated an open-source deep-learning (DL) algorithm called Sybil that accurately predicts long-term lung cancer risk from a single LDCT without the need for human annotation. Sybil has the potential to inform personalized screening strategies among individuals undergoing LDCT, and decrease the risk of both under- and over-screening^21^. However, Sybil was trained on the NLST cohort, which consists of 60% male subjects. Training AI algorithms on biased samples can lead to lower performance in underrepresented groups and Sybil’s performance among males and females has not been compared in a contemporary dataset. Given the known sex differences in lung cancer risk, and the importance of improving risk stratification for females, we aimed to study whether Sybil works equally well among males and females on contemporary LDCT obtained as part of routine clinical care across a large healthcare system.

## Materials and methods

### Participants

With approval from the institutional review board (Mass General Brigham Institutional Review Board), this retrospective study included consecutive subjects who underwent lung cancer screening at Brigham and Women's Hospital (BWH; Boston, USA) or MGH (Boston, USA) between January 1, 2015, and June 30, 2021. The cohort from MGH matches one of the external validation sets used for Sybil^21^. The research was performed in accordance with the Declaration of Helsinki and the Health Insurance Portability and Accountability Act (HIPAA). Informed consent was obtained from all subjects and/or from their legal guardian(s). We chose these cohorts to evaluate Sybil’s performance on contemporary scans obtained as part of routine clinical care. Based on the USPSTF eligibility criteria applicable at the time, patients aged 55 to 80 years with a 30 pack-year history of smoking who were either current smokers or quit smoking within the past 15 years underwent LDCT for lung cancer screening^22^. Participants without clinical follow-up to establish the presence or absence of lung cancer were excluded. We excluded studies obtained after cancer diagnosis and with a slice thickness greater than 2.5 mm to align with Sybil’s training set^21^. Scans with a Lung-RADS score of 0 which indicates that part or all of the lung could not be evaluated were also excluded.

### Data collection

Demographics including sex and smoking history were abstracted from the electronic medical record. LDCT scans were obtained according to standard of care according to the American College of Radiology practice guideline on lung cancer screening^5^. Image acquisition parameters were extracted from the Digital Imaging and Communications in Medicine (DICOM) header of included LDCT scans.

### Lung cancer diagnosis

Participants diagnosed with lung cancer according to the institutional cancer registry within 6 years after the baseline LDCT were considered as having a confirmed lung cancer diagnosis. Those without a lung cancer diagnosis per the cancer registry and one or more negative follow-up screening LDCT scans were considered as not having lung cancer. Screening LDCT was considered negative if the Lung-RADS score was either 1 or 2^23^.

### Image analysis

Sybil is a validated DL algorithm that predicts the future risk of developing lung cancer based on a single LDCT scan^21^. The model aggregates visual information across all three dimensions of the LDCT volume using a 3D convolutional neural network architecture, as previously described^21^.

For each LDCT scan, the algorithm selects the thinnest axial series. Sybil does not require additional clinical data or annotations by a radiologist. Sybil’s output consists of six numbers representing the cumulative lung cancer risk per year over a period of 6 years following each LDCT.

### Statistical analysis

Categorical variables are expressed as frequencies (percentages), while continuous values are expressed as mean ± standard deviation (SD) or median and interquartile range (IQR). Normality of continuous parameters was tested with the Shapiro–Wilk test. To assess Sybil’s performance in the prediction of future lung cancer risk in our cohort stratified by sex, we plotted receiver operating characteristics (ROC) curves for each year up to 6 years following the LDCT. We plotted ROC curves for females and males and compared the area under the curve (AUC) values using DeLong’s test. We computed bootstrapped confidence intervals (CIs) using 5000 resamples after clustering LDCTs by participant. Uno’s concordance (C)-index was computed to express how likely the scan closer to a lung cancer diagnosis had a higher predicted risk in a randomly selected pair of LDCTs. A two-sided p-value of < 0.05 was considered statistically significant for all tests. All analyses were performed using statistical software R (version 4.2.2) and its packages, namely ‘pROC’ (version 1.18.0) and ‘ggplot2’ (version 3.4.2).

## Results

### Baseline characteristics

**Figure 1:**
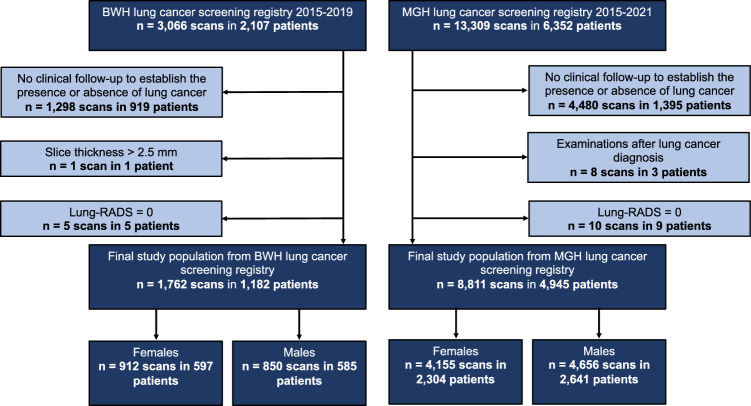
Study flowchart. *BWH* Brigham and Women's Hospital, *MGH* Massachusetts General Hospital.

We obtained 16,375 LDCTs from 8459 consecutive adult subjects (3066 LDCTs from 2107 subjects at BWH and 13,309 LDCTs from 6,352 subjects at MGH). After exclusion, 10,573 LDCTs from 6127 subjects (47.3% female, mean age 64.9 ± 6.2) were analyzed (Fig. 1). 27.6% of the initial study population were excluded due to lack of follow-up. 47.0% of the excluded patients were female and 53.0% were male. Excluded patients were 90.4% white, 3.4% identified as African American, 2.2% as Asian and 4.0% identified with other racial background. Baseline characteristics are summarized in Table 1.

**Table 1:** Characteristics of the 6127 participants of the Lung Cancer Screening Program in a health system from 2015 to 2021, per low-dose chest computed tomography (LDCT).

	Females	Males	p
Number of LDCTs	5067	5506	–
Age (years), mean ± SD	64.9 ± 6.3	64.8 ± 6.2	0.299
Pack-years, median [IQR]	40 [30–50]	40 [35–50]	< 0.001
Race, n (%)			< 0.001
Asian	28 (0.6)	165 (3.0)	
Black or African American	194 (3.8)	201 (3.6)	
White	3999 (78.9)	4388 (79.6)	
Other	149 (2.9)	230 (4.2)	
Unknown	693 (13.7)	531 (9.6)	
Lung-RADS categories per radiology reading for each LDCT, n (%)			0.003
1	841 (16.6)	1058 (19.2)	
2	3389 (66.9)	3515 (63.8)	
3	450 (8.9)	526 (9.6)	
4	387 (7.6)	407 (7.4)	
Future lung cancer, n (%)	186 (3.7)	155 (2.8)	0.015
Number of LDCTs with each year of complete follow-up, defined as having information about a lung cancer diagnosis or a negative follow-up test, n (%)			< 0.001
1	1530 (30.2)	1779 (32.3)	
2	1192 (23.5)	1393 (25.3)	
3	1142 (22.5)	1108 (20.1)	
4	724 (14.3)	753 (13.7)	
5	357 (7.0)	379 (6.9)	
6	124 (2.4)	93 (1.7)	
CT scanner manufacturer, n (%)			0.069
Siemens	1378 (27.2)	1381 (25.1)	
Toshiba	0 (0.0)	1 (0.0)	
General Electric	2500 (49.3)	2791 (50.7)	
Philips	1191 (23.5)	1332 (24.2)	
Scanner kilovoltage peak, n (%)			0.113
100	108 (2.1)	88 (1.6)	
110	3 (0.0)	6 (0.1)	
120	4957 (97.8)	5411 (98.3)	
140	1 (0.0)	0 (0.0)	
Image reconstruction diameter in mm, mean ± SD	394.8 ± 58.8	426.0 ± 49.7	< 0.001
Tube current in mA, median [IQR]	69 [50–92]	80 [55–100]	< 0.001
CTDIvol in mGy, median [IQR]	2.1 [1.7–2.7]	2.7 [1.7–3.0]	< 0.001
Slice thickness in mm, n (%)			< 0.001
0.67	4 (0.1)	0 (0.0)	
1	2551 (50.3)	2704 (49.1)	
1.25	2500 (49.3)	2791 (50.7)	
1.5	5 (0.1)	6 (0.1)	
2	9 (0.2)	4 (0.1)	
Single collimator width in mm, n (%)			< 0.001
0.5	0 (0.0)	1 (0.0)	
0.6	1368 (27.7)	1375 (25.0)	
0.625	3491 (68.9)	3883 (70.5)	
1.2	1 (0.0)	2 (0.0)	
1.25	208 (4.2)	245 (4.4)	
Total collimator width in mm, mean ± SD	46.3 ± 18.4	47.0 ± 18.7	0.062

### Future lung cancer risk prediction in females and males

**Figure 2:**
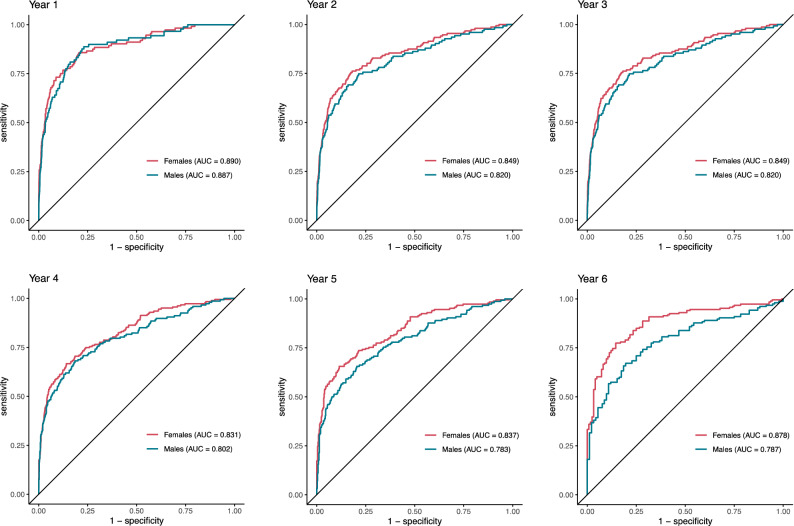
Receiver operating characteristics curves displaying the ability of the Sybil algorithm to predict future lung cancer risk over 6 years following a single low-dose computed tomography scan in 2,901 females and 3,226 males who underwent lung cancer screening between 2015 and 2021. CIs for each curve can be found in Table 2. *AUC* area under the curve.

Lung cancer was diagnosed in 341 (3.2%) cases (3.7% of the females and 2.8% of the males). Sybil predicted the risk of lung cancer with AUCs of 0.89 (95% CI: 0.85–0.93) for females and 0.89 (95% CI: 0.85–0.94) for males at 1 year, 0.85 (95% CI: 0.80–0.90) for females and 0.82 (95% CI: 0.77–0.88) for males at 2 years, 0.83 (95% CI: 0.78–0.88) for females and 0.81 (95% CI: 0.76–0.87) for males at 3 years, 0.83 (95% CI: 0.78–0.88) for females and 0.80 (95% CI: 0.75–0.86) for males at 4 years and 0.84 (95% CI: 0.79–0.89) for females and 0.78 (95% CI: 0.73–0.84) for males at 5 years; all p > 0.05. At 6 years, AUC was 0.88 (95% CI: 0.83–0.93) for females and 0.79 (95% CI: 0.72–0.86) for males, p = 0.009. Detailed data on Sybil’s performance by sex can be seen in Table 2 and Fig. 2.

**Table 2:** Sybil’s future lung cancer predictions per year in 2901 females with 5067 LDCT scans and 3226 males with 5506 LDCT scans between 2015 and 2021.

	Females	Males	p
1-year risk, AUC (95% CI)	0.890 (0.849, 0.934)	0.887 (0.846, 0.936)	0.915
2-year risk, AUC (95% CI)	0.849 (0.803, 0.898)	0.820 (0.766, 0.875)	0.320
3-year risk, AUC (95% CI)	0.827 (0.777, 0.878)	0.809 (0.756, 0.867)	0.549
4-year risk, AUC (95% CI)	0.831 (0.784, 0.877)	0.802 (0.749, 0.857)	0.308
5-year risk, AUC (95% CI)	0.837 (0.792, 0.886)	0.783 (0.727, 0.842)	0.067
6-year risk, AUC (95% CI)	0.878 (0.829, 0.930)	0.787 (0.715, 0.860)	0.009
C-index (95% CI)	0.834 (0.788, 0.881)	0.814 (0.767, 0.866)	

*AUC* area under the curve, *C-Index* concordance index.

When comparing Sybil’s risk prediction between the two centers, the performance was significantly better at BWH at 1 year (0.97 [95% CI: 0.94–0.99]) than at MGH (0.86 [95% CI: 0.82–0.90], p < 0.001). From the second year of follow-up onward, this difference in performance was not statistically significant (Table 3). Additional details regarding Sybil’s ability to predict lung cancer by center are reported in Supplementary Table 1.

**Table 3:** Sybil’s risk prediction for future lung cancer risk in 2901 females with 5067 LDCT scans and 3226 males with 5506 LDCT scans, by hospital.

	BWH and MGH	BWH	MGH	p
1-year risk, AUC (95% CI)	0.886 (0.856, 0.919)	0.965 (0.943, 0.998)	0.859 (0.819, 0.904)	< 0.001
2-year risk, AUC (95% CI)	0.835 (0.800, 0.873)	0.881 (0.821, 0.953)	0.816 (0.773, 0.861)	0.050
3-year risk, AUC (95% CI)	0.817 (0.78, 0.853)	0.850 (0.783, 0.926)	0.794 (0.748, 0.843)	0.101
4-year risk, AUC (95% CI)	0.816 (0.781, 0.852)	0.835 (0.767, 0.909)	0.786 (0.741, 0.833)	0.146
5-year risk, AUC (95% CI)	0.811 (0.774, 0.849)	0.812 (0.740, 0.887)	0.780 (0.732, 0.830)	0.367
6-year risk, AUC (95% CI)	0.838 (0.796, 0.882)	0.796 (0.714, 0.877)	–	–
C-index (95% CI)	0.823 (0.790, 0.859)	0.844 (0.781, 0.909)	0.81 (0.769, 0.852)	

*AUC* area under the curve, *BWH* Brigham and Women's Hospital, *C-Index* concordance index, *MGH* Massachusetts General Hospital.

## Discussion

The validated DL algorithm called Sybil accurately predicted future lung cancer risk in both females and males with AUCs of 0.89 (95% CI: 0.85–0.93) for females and 0.89 (95% CI: 0.85–0.94) for males at 1 year. For long-term lung cancer risk prediction at 6 years, Sybil performed better in females than in males with AUCs of 0.88 (95% CI: 0.83–0.93) for females and 0.79 (95% CI: 0.72–0.86) for males. This study provides a validation of the prediction of sex-stratified lung cancer incidence up to 6 years in a large cohort of participants who recently underwent lung cancer screening as part of standard of care.

The role of artificial intelligence (AI) continues to expand in medicine. However, the utility of AI-generated predictions is largely dependent on the quality and diversity of the training datasets^24^. Females have been underrepresented in clinical trials, including in the NLST^25^. The 2020 report on ‘Artificial Intelligence and Gender Equality’ by UNESCO (United Nations Educational, Scientific and Cultural Organization) calls for increased efforts to address the potential for sex inequities in AI^26^. Given the possibility for biases to be introduced during the development of DL models, careful evaluation is warranted using contemporary data collected from the clinical setting in which deployment is considered^27^. If uncovered, biases can be addressed by re-training and adapting the algorithm, thereby preventing future digital systems to the perpetuate disparities of the past^28^.

The current study adds to prior work in several ways. Sybil was developed on a 60% male population using LDCT scans obtained almost two decades ago as part of a clinical trial while our study population consisted of more than 10,500 contemporary LDCT scans from participants who underwent lung cancer screening LDCT as standard of care since 2015. The percentage of males in the current study is a more balanced 53% and reflects current clinical practice in our health system. In the current study, Sybil performed equally well at predicting lung cancer risk in both sexes in years 1–5 and demonstrated significantly better performance in risk prediction among females at 6 years. The difference in performance at 6 years is hypothesis generating and might reflect Sybil’s capability to detect early signs of tumorigenesis. A prior study showed that lung cancer prevalence was nearly twice as high in females compared to males of similar age and smoking history^29^. Consistent with this finding, the overall incidence of lung cancer in our study population was higher in females than in males (3.7% in females vs. 2.8% in males). These results suggest that lung cancer may progress more slowly in females and might contribute to Sybil’s better performance in females at 6 years.

Lung cancer remains the leading cause of cancer mortality in the United States in both sexes. However, females have different risk profiles as compared to males and over the last decades, the incidence of lung cancer has decreased far more slowly in females compared to males^16^. Moreover, lung cancer in people who never smoked is increasing world-wide, especially among women^13^. The 2021 USPSTF lung cancer screening eligibility criteria lowered the age at which one can access screening from 55 to 50 years and reduced the requisite smoking pack-years from 30 to 20^5^. These changes should ideally have reduced sex disparities in eligibility by increasing the number of females eligible for lung cancer screening. However, data suggest that while more women can now access lung screening LDCT, sex disparities in eligibility still persist^14^. Given the potential inaccuracy of widely used clinical risk scores when applied to females, identifying more accurate risk stratification tools is particularly important in females, as it is in racially and ethnically minoritized groups.

DL-based prediction systems for lung cancer screening have shown promise in assisting radiologists with early detection and risk assessment^2,30–32^. We anticipate that DL-based algorithms will eventually be integrated into clinical practice, which raises medicolegal concerns related to liability, accuracy, patient consent, and data privacy. Addressing potential biases in DL-based algorithms rigorously, establishing clear user guidelines, and continuously evaluating these systems are essential steps to mitigate these challenges and promote the integration of DL-based prediction systems into patient management pathways.

It is important to acknowledge that our cohort predominantly comprised participants identified as White, constituting approximately 80% of the participants both in males and females. This raises concerns about the generalizability of our results to more diverse populations, as both race/ethnicity and socioeconomic status disparities are associated with lung cancer risk and might affect Sybil’s performance^33,34^. Studies are required to assess Sybil’s performance in more diverse populations. A considerable proportion of patients were excluded from the analysis due to the lack of clinical follow-up. While no imbalance was detected between the included and excluded groups in terms of sex and race/ethnicity, there may have unmeasured differences in these groups.

There are several limitations to this study. First, this was a retrospective study relying on routinely collected clinical data which may be subject to misclassification. Second, we did not evaluate the impact of other demographic features or the possible mediating and compounding impacts of socioeconomic status or gender, which may affect lung cancer risk. Third, this experiment was conducted using data from a single health system with limited racial and ethnic diversity. This study was limited to participants who qualified for lung cancer screening under the 2013 USPSTF criteria since CT scans were obtained between 2015 and 2021, prior to the release of the 2021 USPSTF criteria. Another limitation of our study is the limited follow-up time, which mirrors the current state of lung cancer screening.

In summary, our results did not reveal sex differences in the performance of an open access DL algorithm that predicts lung cancer risk based on a single LDCT, suggesting that Sybil can accurately predict future lung cancer risk in females and males and there is no need for retraining. For predicting long-term lung cancer risk at 6 years, Sybil performs better in females than in males. Pending validation in a more racially diverse populations and confirmation of benefit in prospective trials, both of which are underway, Sybil may eventually be deployed in clinical settings. For example, Sybil could potentially be used to risk-stratify individuals following their annual screening LDCT, thereby enabling the extension of screening interval for low-risk individuals.

### Supplementary Information

Supplementary Table S1.

## Data Availability

The datasets used and analyzed during the current study are available from the corresponding author on reasonable request.

## Abbreviations

AI: Artificial intelligence
AUC: Area under the curve
CI: Confidence interval
IQR: Interquartile range
LDCT: Low-dose computed tomography
Lung-RADS: Lung Reporting and Data System
ROC: Receiver operating characteristics
UNESCO: United Nations Educational, Scientific and Cultural Organization
