# Supplementary material for: Role of sex in lung cancer risk prediction based on single low-dose chest computed tomography
Source: Sci Rep. 2023 Oct 30;13:18611. doi: 10.1038/s41598-023-45671-6 (PMC10616081; doi:10.1038/s41598-023-45671-6)
Supplement: Supplementary file 1 — Supplementary Table S1. [file 41598_2023_45671_MOESM1_ESM.docx]

## Supplementary Table 1.

Sybil’s Future Lung Cancer Predictions per Year in 2,901 Females with 5,067 LDCT scans and 3,226 Males with 5,506 LDCT scans, stratified by hospital

|  | BWH | | | MGH | | |
| --- | --- | --- | --- | --- | --- | --- |
|  | Females | Males | p | Females | Males | p |
| 1-Year Risk, AUC (95%CI) | 0.973 (0.950, 1.013) | 0.955 (0.924, 1.004) | 0.490 | 0.862 (0.809, 0.92) | 0.862 (0.808, 0.926) | 0.993 |
| 2-Year Risk, AUC (95%CI) | 0.861 (0.776, 0.969) | 0.894 (0.829, 0.977) | 0.574 | 0.836 (0.783, 0.895) | 0.793 (0.725, 0.863) | 0.211 |
| 3-Year Risk, AUC (95%CI) | 0.842 (0.755, 0.944) | 0.847 (0.759, 0.939) | 0.943 | 0.799 (0.738, 0.864) | 0.793 (0.727, 0.862) | 0.845 |
| 4-Year Risk, AUC (95%CI) | 0.839 (0.753, 0.935) | 0.820 (0.725, 0.919) | 0.759 | 0.789 (0.728, 0.855) | 0.784 (0.717, 0.852) | 0.876 |
| 5-Year Risk, AUC (95%CI) | 0.829 (0.737, 0.928) | 0.776 (0.681, 0.875) | 0.416 | 0.787 (0.725, 0.853) | 0.776 (0.705, 0.850) | 0.764 |
| 6-Year Risk, AUC (95%CI) | 0.813 (0.714, 0.928) | 0.751 (0.637, 0.873) | 0.388 | - | - | - |
| C-Index, (95%CI) | 0.846 (0.746, 0.954) | 0.832 (0.756, 0.921) |  | 0.819 (0.766, 0.878) | 0.802 (0.744, 0.863) |  |

Abbreviations: AUC=area under the curve; BWH=Brigham and Women’s Hospital; C-Index=Concordance index; MGH=Massachusetts General Hospital.
